# Lymph node metastasis regulation by peritumoral tonsillar tissue mitochondria-related pathway activation in oropharyngeal cancer

**DOI:** 10.1371/journal.pone.0299750

**Published:** 2024-02-28

**Authors:** Naohiro Wakisaka, Makiko Moriyama-Kita, Satoru Kondo, Eiji Kobayashi, Takayoshi Ueno, Yosuke Nakanishi, Kazuhira Endo, Hisashi Sugimoto, Tomokazu Yoshizaki

**Affiliations:** 1 Department of Otorhinolaryngology, NHO Kanazawa Medical Center, Kanazawa, Ishikawa, Japan; 2 Division of Otorhinolaryngology and Head and Neck Surgery, Graduate School of Medical Science, Kanazawa University, Kanazawa, Ishikawa, Japan; University of Nebraska-Lincoln, UNITED STATES

## Abstract

Immune-related gene expression profiles of peritumoral tonsillar tissues are modified by oropharyngeal cancer (OPC) nodal status. This study explored immunometabolism and immune cell count alterations in peritumoral tonsillar tissue according to OPC nodal status. Microarray data analysis of 27 peritumoral tonsillar tissue samples, using a newly generated mitochondrial metabolism-related gene set comprised of 948 genes, detected 228 differentially expressed genes (DEGs) (206 up- and 22 downregulated) in metastasis-negative cases compared to metastasis-positive ones. REACTOME pathway analysis of the 206 upregulated genes revealed the Toll-like receptor 4 cascade were most enriched. Immune cell proportion analysis using the CIBERSORTx algorithm revealed a significantly higher rate of naïve B cells, but lower rates of regulatory T cells and resting natural killer cells in metastasis-negative cases. Digital spatial profiling of the 6 OPC tissues detected 9 DEGs in the lymphoid regions, in contrast, no DEGs were identified in tumor regions according to nodal status. Cancer cell nests and pair matched normal epithelia mitochondrial DNA (mtDNA) from 5 OPC tissues were analyzed by next generation sequencing for variant detection. However, no significant mtDNA variation was found. This study identified mitochondria-related immune cell transcriptional programs and immune cell profiles associated with OPC lymphatic spread in peritumoral tonsil tissue, further evaluation of which will elucidate targetable immune mechanisms associated with OPC lymphatic dissemination.

## Introduction

The increasing prevalence of oropharyngeal cancer (OPC) is largely due to rising numbers of human papillomavirus (HPV)-positive cancers [1]. The most frequent treatment modality for OPCs is radiotherapy, with or without chemotherapy [2]. The presence of HPV in OPCs is associated with an improved therapeutic response relative to HPV-negative counterparts [3,4].

Tumor microenvironments (TMEs) comprise of a variety of cells, including proliferating and non-proliferating tumor cells, as well as stromal cells, such as endothelial cells and cancer associated fibroblasts, and immune cells. The immune system plays an important role in detecting and eliminating tumor cells, thereby preventing tumor dissemination. Recently, substantial numbers of findings concerning tumor immunometabolism were reported, including head and neck cancers such as OPCs. These findings relate immune cell metabolism and function changes [5,6]. It is well established that tumor cells favor aerobic glycolysis over oxidative phosphorylation (OXPHOS) even in abundant oxygen (the Warburg effect). In TMEs, aerobic glycolysis is a central metabolic program associated with the activation of innate (dendritic cells, macrophages, and neutrophils) and adaptive (CD8^+^ and CD4^+^ T cells, B cells) immune cells, the limitation of which compromises effector functions and the anti-tumor effects of these cells [7]. Therefore, in TMEs where aerobic glycolysis is limited by metabolic competition between tumor and immune cells, activating alternative immunometabolic pathways to aerobic glycolysis, such as mitochondria-related pathways, are necessary to regulate tumor progression pathways, such as the lymphatic dissemination of the disease.

Classically, efficient adaptive immune responses against cancer occur in the secondary lymphoid organs (SLOs) [8,9]. Dendritic cells (DCs) migrate from the tumor site to the SLOs, wherein major histocompatibility complex molecule-peptide complexes are presented to CD4^+^ T and CD8^+^ T cells by mature DCs. B cells are activated in the SLOs upon primary follicle antigen binding and receive help from CD4^+^ T cells, proliferating to form a secondary follicle that progressively becomes a germinal center. These steps allow lymphocyte proliferation and differentiation into effector T cells and memory B cells that migrate into the tumor site and lead to the destruction of tumor cells. Palatine tonsils are SLOs located in the oropharynx that are important in host defense against pathogens invading the upper respiratory tract [10]. Therefore, in OPCs, the peritumoral tonsillar tissue immunologic processes are likely crucial for effective anti-tumor immune responses by providing primary tumor sites with activated immune cells. Previously, we reported immune-related transcription profiles of peritumoral tonsillar tissues analyzed according to OPC nodal status [11]. As expected, in the peritumoral tonsillar tissues, large proportions of immune-related genes were differentially expressed between metastasis-negative and metastasis-positive cases. These findings suggest peritumoral tonsillar tissue as a potential target to investigate immune mechanism associations with lymphatic dissemination of the disease in OPCs. However, detailed immunological profiles according to OPC nodal status, such as changes in immunometabolism and immune cell types, are yet to be elucidated.

In this study, bioinformatic methods, including REACTOME pathway analysis and the CIBERSORTx algorithm, were applied to analyze immunometabolism and immune cell count alterations in the peritumoral tonsillar tissues of OPCs according to nodal status. The data and analytical approaches in this study are expected to assist in elucidating immune system roles and mechanisms in the metastatic dissemination of OPCs.

## Materials and methods

### Patient population

A total of 48 oropharyngeal primary tumor radical resections performed for OPCs originating at the palatine tonsil were selected from the pathology files of Kanazawa University from 2012 to 2020. The authors had access to identifying information for individual participants during or after data collection. Staging at diagnosis was according to the seventh edition of TNM classification of malignant tumors of the Union Internationale Contre le Cancer [12]. Ten cases were excluded from analysis; one with positive surgical margin at the primary site followed by postoperative radiotherapy, one with recurrence at the primary site, and 8 cases without sufficient tonsillar tissue for subsequent analysis. Thus, in this study, 38 out of 48 patients were enrolled and subdivided into three cohorts based on sample assessment using microarray analysis (Cohort 1), GeoMx whole transcriptome atlas (WTA) analysis (Cohort 2), or mitochondrial DNA (mtDNA) analysis (Cohort 3). The detailed characteristics of the 38 patients with OPC are listed in Table 1. Following primary surgery, 8 patients received adjuvant radiation treatment concomitant with and without chemotherapy, due to multiple metastasis-positive lymph nodes.

**Table 1:** Clinical characteristics of the 38 patients with oropharyngeal cancer included in the study.

Factors		Cohort 1 (n = 27)	Cohort 2 (n = 6)	Cohort 3 (n = 5)
**Age**	**Median**	62 (43–88)	64.5 (45–84)	65 (43–85)
**Sex**	**Male**	22	6	4
	**Female**	5	0	1
**HPV**	**Positive**	23	4	3
	**Negative**	4	2	2
**Stage**	**I**	23	5	1
	**II**	4	0	1
	**III**	0	0	1
	**IVA**	0	1	2
**T classification**	**T1**	11	3	1
	**T2**	14	3	3
	**T3**	2	0	1
**Lymph Node Metastasis**	**Negative**	7	3	2
	**Positive**	20 (3*)	3 (0*)	3
**M classification**	**M0**	27	6	5

Cohort 1 (n = 27) samples were subject to microarray analysis. Cohort 2 (n = 6) samples were analyzed using GeoMx whole transcriptome atlas. Cohort 3 (n = 5) samples were evaluated using mitochondrial DNA next generation sequencing. *, Number of cases with delayed lymph node metastasis in each cohort. HPV, human papillomavirus.

For Cohort 1, 27 patients were enrolled, of which 7 were lymph node metastasis-negative with the other 20 metastasis-positive. For Cohort 2, 6 patients were enrolled consisting of 3 lymph node metastasis-negative, and 3 metastasis-positive cases. For Cohort 3, 5 patients were enrolled, with 2 lymph node metastasis-negative patients and 3 metastasis-positive. The classification of lymph node metastasis-positive includes all patients with lymph node metastasis at any stage of the clinical course, including diagnosis, post-operative pathological diagnosis, and during the follow-up period. This data was analyzed on the 3rd of October, 2023.

This study was approved by the Bioethics Committee of Kanazawa University (No. 2016–033). Written informed consent was obtained from all patients enrolled in this study.

### Microarray and computational analysis

Total RNA from formalin-fixed paraffin-embedded (FFPE) blocks was isolated using RNeasy FFPE Kit (QIAGEN, Tokyo, JAPAN). RNA quality and quantity were evaluated using a NanoDrop ND-1000 spectrophotometer (Thermo Fisher, Wilmington, DE). For Cohort 1, total RNA samples were prepared from peritumoral tonsillar tissues (S1A Fig), excluding the tumor area. These samples were macro-dissected from whole FFPE tissues on a glass slide.

Reverse transcription and amplification of 100 pg of high-quality total RNA was performed using the GeneChip WT Pico kit (Applied Biosystems, Foster City, CA). Following linear amplification, the obtained complementary RNA was converted to biotinylated sense-strand cDNA targets for hybridization. After hybridization in a GeneChip Hybridization Oven, the array was washed, stained, and scanned. This data was then analyzed using the Transcriptome Analysis Console 4.0.2.15 Software (Thermo Fisher, Wilmington, DE). The expression data was processed using a robust multichip average normalization algorithm. The datasets (GSE228432) are available from the Gene Expression Omnibus database (https://www.ncbi.nlm.nih.gov/geo/).

### Generating a mitochondria-related gene set and REACTOME pathway enriched analysis

A total of 51 mitochondria-related gene sets were selected using the Gene Set Enrichment Analysis (GSEA) database (https://www.gsea-msigdb.org/gsea/index.jsp), including 13 mitochondria metabolism-related, 10 reactive oxygen species (ROS) metabolism-related, 8 tricarboxylic acid (TCA) cycle metabolism-related, 11 fatty acid metabolism-related, and 9 glycolysis metabolism-related gene sets. Detailed information of the 51 gene sets in total from the GSEA database are shown in Table 2.

**Table 2:** The detailed mitochondria, ROS, TCA cycle, Fatty Acid, and Glycolysis metabolism-related gene sets from the GSEA database. (https://www.gsea-msigdb.org/gsea/index.jsp).

Metabolism	Gene Sets	NOM p-value	FDR q-value
**Mitochondria**	WP_MITOCHONDRIAL_GENE_EXPRESSION	0.01458	0.10336
	REACTOME_TRANSCRIPTIONAL_ACTIVATION_OF_MITOCHONDRIAL_BIOGENESIS	0.02362	0.05970
	BIOCARTA_MITOCHONDRIA_PATHWAY	0.03462	0.05400
	MOOTHA_MITOCHONDRIA	0.03226	0.07709
	WP_NAD_METABOLISM_IN_ONCOGENEINDUCED_SENESCENCE_AND_MITOCHONDRIAL_DYSFUNCTIONASSOCIATED_SENESCENCE	0.05714	0.13063
	GALLUZZI_PERMEABILIZE_MITOCHONDRIA	0.09658	0.12394
	HP_MITOCHONDRIAL_RESPIRATORY_CHAIN_DEFECTS	0.04848	0.11118
	HP_DECREASED_ACTIVITY_OF_MITOCHONDRIAL_COMPLEX_I	0.11156	0.09728
	HP_DECREASED_ACTIVITY_OF_MITOCHONDRIAL_COMPLEX_IV	0.13828	0.09321
	HP_DECREASED_ACTIVITY_OF_MITOCHONDRIAL_COMPLEX_II	0.10385	0.12332
	REACTOME_MITOCHONDRIAL_FATTY_ACID_BETA_OXIDATION	0.11492	0.12832
	REACTOME_MITOCHONDRIAL_FATTY_ACID_BETA_OXIDATION_OF_SATURATED_FATTY_ACIDS	0.42442	0.36398
	HP_DECREASED_ACTIVITY_OF_MITOCHONDRIAL_COMPLEX_III	0.61031	0.50389
**ROS**	GOBP_CELLULAR_RESPONSE_TO_REACTIVE_OXYGEN_SPECIES	0.02692	0.11247
	GOBP_REGULATION_OF_REACTIVE_OXYGEN_SPECIES_METABOLIC_PROCESS	0.00994	0.07691
	GOBP_POSITIVE_REGULATION_OF_REACTIVE_OXYGEN_SPECIES_METABOLIC_PROCESS	0.02930	0.06979
	GOBP_REGULATION_OF_RESPONSE_TO_REACTIVE_OXYGEN_SPECIES	0.02918	0.05827
	GOBP_NEGATIVE_REGULATION_OF_REACTIVE_OXYGEN_SPECIES_METABOLIC_PROCESS	0.01875	0.05558
	GOBP_REACTIVE_OXYGEN_SPECIES_METABOLIC_PROCESS	0.04600	0.08963
	GOBP_REGULATION_OF_REACTIVE_OXYGEN_SPECIES_BIOSYNTHETIC_PROCESS	0.07739	0.11749
	HALLMARK_REACTIVE_OXYGEN_SPECIES_PATHWAY	0.15030	0.11076
	REACTOME_DETOXIFICATION_OF_REACTIVE_OXYGEN_SPECIES	0.23340	0.14559
	GOBP_REACTIVE_OXYGEN_SPECIES_BIOSYNTHETIC_PROCESS	0.15126	0.15362
**TCA Cycle**	REACTOME_THE_CITRIC_ACID_TCA_CYCLE_AND_RESPIRATORY_ELECTRON_TRANSPORT	0.03629	0.10807
	REACTOME_CITRIC_ACID_CYCLE_TCA_CYCLE	0.11440	0.29769
	WP_TCA_CYCLE_AKA_KREBS_OR_CITRIC_ACID_CYCLE	0.12451	0.19894
	REACTOME_PYRUVATE_METABOLISM_AND_CITRIC_ACID_TCA_CYCLE	0.12676	0.19284
	MOOTHA_TCA	0.19643	0.22019
	WP_TCA_CYCLE_NUTRIENT_UTILIZATION_AND_INVASIVENESS_OF_OVARIAN_CANCER	0.14199	0.22237
	KEGG_CITRATE_CYCLE_TCA_CYCLE	0.26285	0.20763
	WP_TCA_CYCLE_AND_DEFICIENCY_OF_PYRUVATE_DEHYDROGENASE_COMPLEX_PDHC	0.26494	0.18208
**Fatty Acid**	HALLMARK_FATTY_ACID_METABOLISM	0.04564	0.26401
	GOBP_FATTY_ACID_HOMEOSTASIS	0.05510	0.23541
	REACTOME_MITOCHONDRIAL_FATTY_ACID_BETA_OXIDATION	0.11465	0.31695
	GOBP_FATTY_ACID_CATABOLIC_PROCESS	0.13570	0.24089
	KEGG_FATTY_ACID_METABOLISM	0.15351	0.27133
	REACTOME_FATTY_ACID_METABOLISM	0.20316	0.38928
	GOBP_FATTY_ACID_DERIVATIVE_BIOSYNTHETIC_PROCESS	0.26016	0.33399
	GOBP_FATTY_ACID_METABOLIC_PROCESS	0.22883	0.34210
	GOBP_CELLULAR_RESPONSE_TO_FATTY_ACID	0.66239	0.70080
	FATTY_ACID_OXIDATION	0.62395	0.63094
	GOBP_FATTY_ACID_DERIVATIVE_METABOLIC_PROCESS	0.72198	0.66716
**Glycolysis**	REACTOME_GLYCOLYSIS	0.13673	0.68098
	HALLMARK_GLYCOLYSIS	0.11614	0.35265
	WP_GLYCOLYSIS_IN_SENESCENCE	0.14794	0.24128
	REACTOME_REGULATION_OF_GLYCOLYSIS_BY_FRUCTOSE_2_6_BISPHOSPHATE_METABOLISM	0.27925	0.45644
	BIOCARTA_GLYCOLYSIS_PATHWAY	0.49109	0.62283
	WP_COMPUTATIONAL_MODEL_OF_AEROBIC_GLYCOLYSIS	0.58383	0.74711
	WP_GLYCOLYSIS_AND_GLUCONEOGENESIS	0.72809	0.73962
	WP_HIF1A_AND_PPARG_REGULATION_OF_GLYCOLYSIS	0.71032	0.76974
	KEGG_GLYCOLYSIS_GLUCONEOGENESIS	0.91897	0.79835

The significantly enriched gene sets are highlighted in yellow. FDR q-value, false discovery rate q-value; GSEA, Gene Set Enrichment Analysis; NOM p-value, nominal p-value; ROS, reactive oxygen species; TCA Cycle, tricarboxylic acid cycle.

GSEA was performed for Cohort 1 samples using GSEA software version 4.1.0 (GSEA; Broad Institute, Cambridge, MA) with the 51 gene sets. Using the conventional thresholds of significant nominal (NOM) p-value (< 0.05) and false discovery rate (FDR) q-value (< 0.25), according to established GSEA criteria [13], significant differences in gene set enrichment between metastasis-negative and metastasis-positive cases were observed. Specifically, 5 out of 13 mitochondria metabolism-related, 6 out of 10 ROS metabolism-related, and 1 out of 8 TCA cycle metabolism-related gene sets were significantly enriched in metastasis-negative cases. In contrast, no fatty acid metabolism-related or glycolysis metabolism-related gene sets were significantly enriched. The 948 genes were listed as a newly generated mitochondria-related gene set by combining the genes from the above mentioned 12 significantly enriched gene sets (S1 Table). For Cohort 1, the 948 genes were used to detect differentially expressed genes (DEGs) between 7 metastasis-negative and 20 metastasis-positive cases. For Cohort 1, the 948 genes were also assessed for DEGs according to HPV-status. The DEG selection criterium was an adjusted p-value of < 0.05 and a log_2_FC > 0.

**Fig 1:**
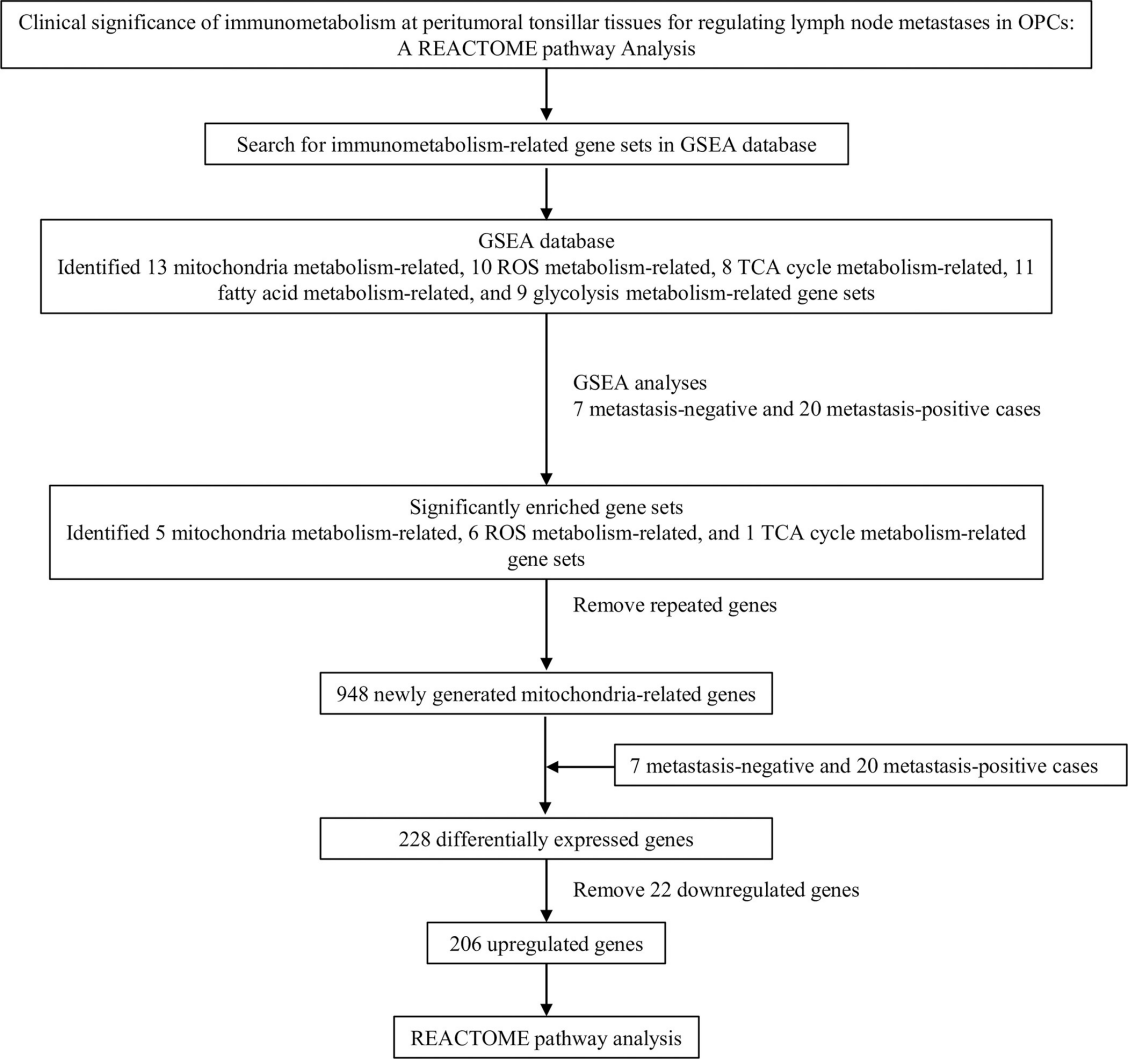
The overall REACTOME pathway analysis workflow of the current study. GSEA, Gene Set Enrichment Analysis; OPCs, Oropharyngeal cancers: ROS, reactive oxygen species; TCA, tricarboxylic acid.

For Cohort 1, ClueGO, a widely used Cytoscape plugin allowing visualization of nonredundant biological terms for large clusters of DEGs in a grouped network, was used to decipher the functionally grouped REACTOME pathway using Cytoscape software version 3.10.1 (https://cytoscape.org). The REACTOME pathway analysis workflow is shown in Fig 1.

### GeoMx WTA analyses

The GeoMx Digital Spatial Profiler (NanoString Technologies, Seattle, WA) performs high-plex profiling at protein and RNA levels, providing spatial and temporal assessment of tumors in frozen or FFPE limited tissue samples [14]. Using this platform, WTA evaluates over 18,000 protein-coding genes based on the human gene nomenclature committee database cross-referenced with available mRNA sequences in the National Center for Biotechnology’s information RefSeq database. The whole human transcriptome is measured in each region of interest (ROI) to identify biological changes at specific tissue locations. For ROI selection, morphology markers (PanCK, CD45, and SMA) were used to distinguish tumor cells, immune cells, and mesenchymal cells, respectively.

**Fig 2:**
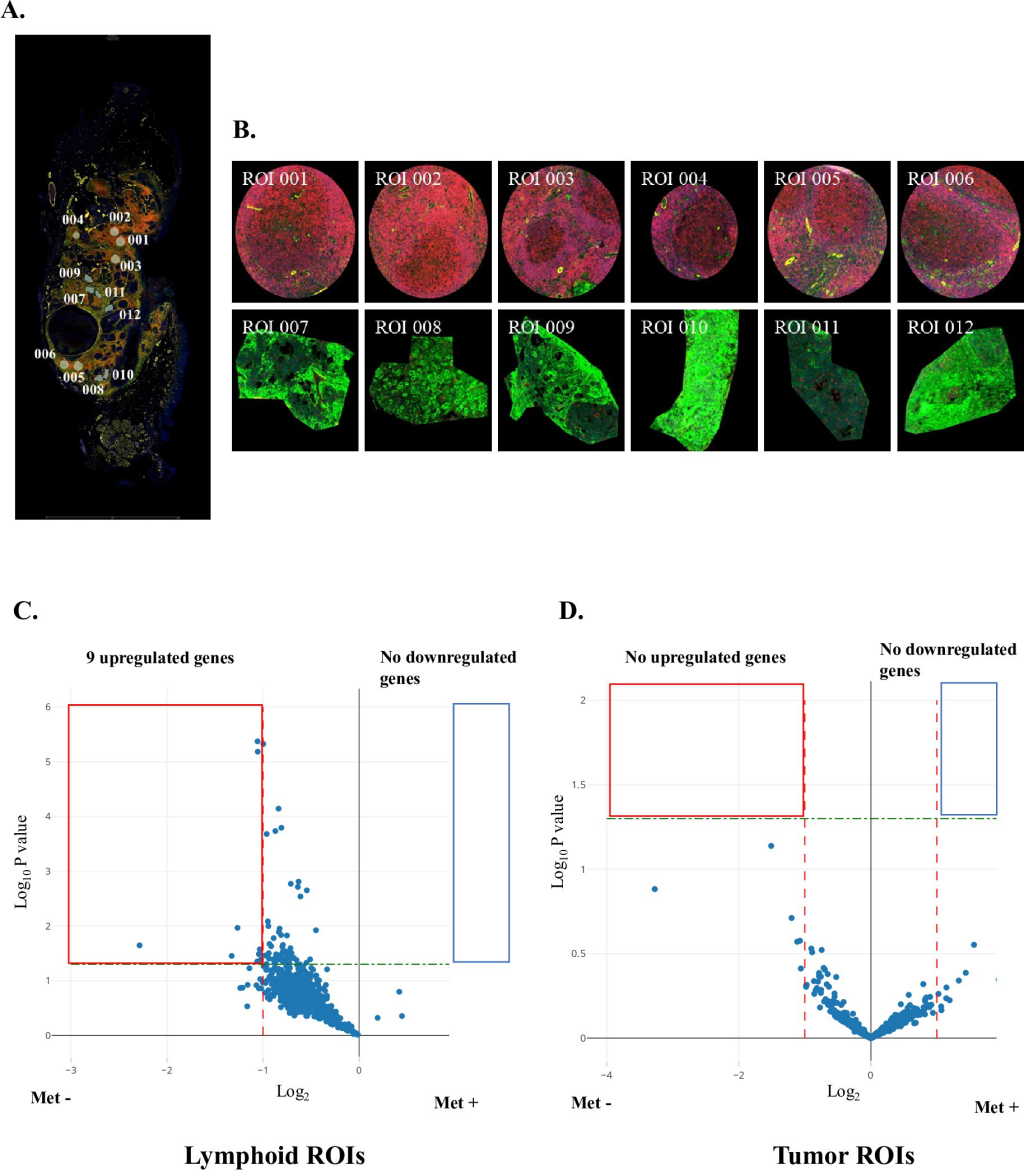
GeoMx whole transcriptome atlas analysis (WTA) of a resected transoral oropharyngectomy surgical specimen. **A:** Low-power fluorescence view (x 100) of the specimen stained for PanCK (green), CD45 (red), SMA (yellow), and DNA (blue). Regions of interest (ROIs) 001–006 outlined in white are lymphoid areas within the specimen, while ROIs 007–012 are tumor areas. **B:** High-power views (x 400) of ROIs 001–006, lymphoid areas, and ROIs 007–012, tumor areas, are shown in each panel. **C** and **D:** Volcano plots of differentially expressed genes in metastasis-negative (Met-) and metastasis-positive (Met+) oropharyngeal cancers (OPCs) in WTA analysis. Blue dots in the **red** and **blue boxed** regions indicate up- and downregulated genes in Met- cases compared with Met+ cases. Significance was defined as |log_2_| of fold-change > 1 and -log_10_ *P* > 1.3. The **red** and **green dashed lines** indicate |log_2_| of fold-change = 1 and -log_10_ *P* = 1.3, respectively. **C:** Analysis of lymphoid ROIs adjacent to OPC tumor nests reveals a total of 9 upregulated genes. **D:** Tumor ROI analysis revealed no differentially expressed genes.

The 6 samples of Cohort 2 were evaluated by GeoMx WTA analysis. Fig 2A and 2B demonstrate fluorescent staining and ROI selection for a representative OPC tissue sample. For each sample, 12 ROIs were selected, of which 6 were lymphoid regions, and 6 were tumor regions. A total of 36 ROIs in the lymphoid and tumor regions were classified as metastasis-negative or metastasis-positive, respectively.

A linear mixed model was applied to detect DEGs between the two groups using the generated mitochondria-related gene set comprised of the 948 genes described above. Statistical significance was defined as a |log_2_| of fold change > 1 and a -log_10_ p-value > 1.3.

### mtDNA analyses for variant detection by next generation sequencing (NGS)

Laser captured microdissection was performed using a PALM MicroBeam (Carl Zeiss AG, Aalen, Germany) for the Cohort 3 samples. Cancer cell nests and matched normal epithelia were microdissected from each hematoxylin and eosin stained FFPE tissue slide of the oropharyngeal primary tumor. DNA extraction from the microdissected tissue was performed using an AllPrep DNA/RNA FFPE kit (QIAGEN, Venlo, Netherland) for mtDNA analyses.

Cancer cell nest mtDNA was analyzed by NGS for variant detection. To exclude constitutional polymorphism variants, mtDNA from paired cancer nests and their matched normal epithelia were evaluated for all Cohort 3 samples. NGS libraries for variant detection were constructed with the QIAseq Targeted DNA Panel-Human Mitochondria Panel (DHS-105Z) (QIAGEN, Venlo, Netherland) covering the whole mitochondrial genome. This NGS panel is based on the Association for Molecular Pathology guidelines, and uses unique molecular identifiers to better differentiate NGS artifacts from real mutations at very low allele fractions. Final mtDNA library concentrations were 10 pM. Reads were aligned to srID from the Single Nucleotide Polymorphism Database (dbSNP) (https://ncbi.nlm.nih.gov/snp/).

### Cell Deconvolution analyses

Relative immune cell proportions analyses were performed to further investigate peritumoral tonsillar tissue immunity differences between metastasis-negative and metastasis-positive patients. CIBERSORTx (https://cibersortx.stanford.edu) was used to estimate the relative proportion of 22 infiltrating immune cell types in OPC peritumoral tonsillar tissue. Each tonsillar tissue sample from microarray analyses was deconvoluted, and the putative proportion of infiltrating immune cell types obtained. The LM22 signature matrix, a validated leukocyte gene signature matrix containing 547 genes, was used to distinguish 22 human immune cell subsets [15]. The immune cell proportion differences between metastasis-negative and metastasis-positive cases were then assessed by the Mann-Whitney U test with a p-value < 0.05 considered significant.

## Results

### Detection of DEGs and REACTOME pathway analysis using the newly generated mitochondria-related gene set

From the peritumoral tonsillar tissues of Cohort 1, 27 RNA samples were prepared for expression level analyses of the 948 genes from the newly generated mitochondria-related gene set as described in Materials and Methods. For the 948 genes, 228 genes were differentially expressed (206 upregulated and 22 downregulated genes) in metastasis-negative cases compared with metastasis-positive ones (S1B Fig). None of the 948 genes were differentially expressed according to HPV-status.

**Fig 3:**
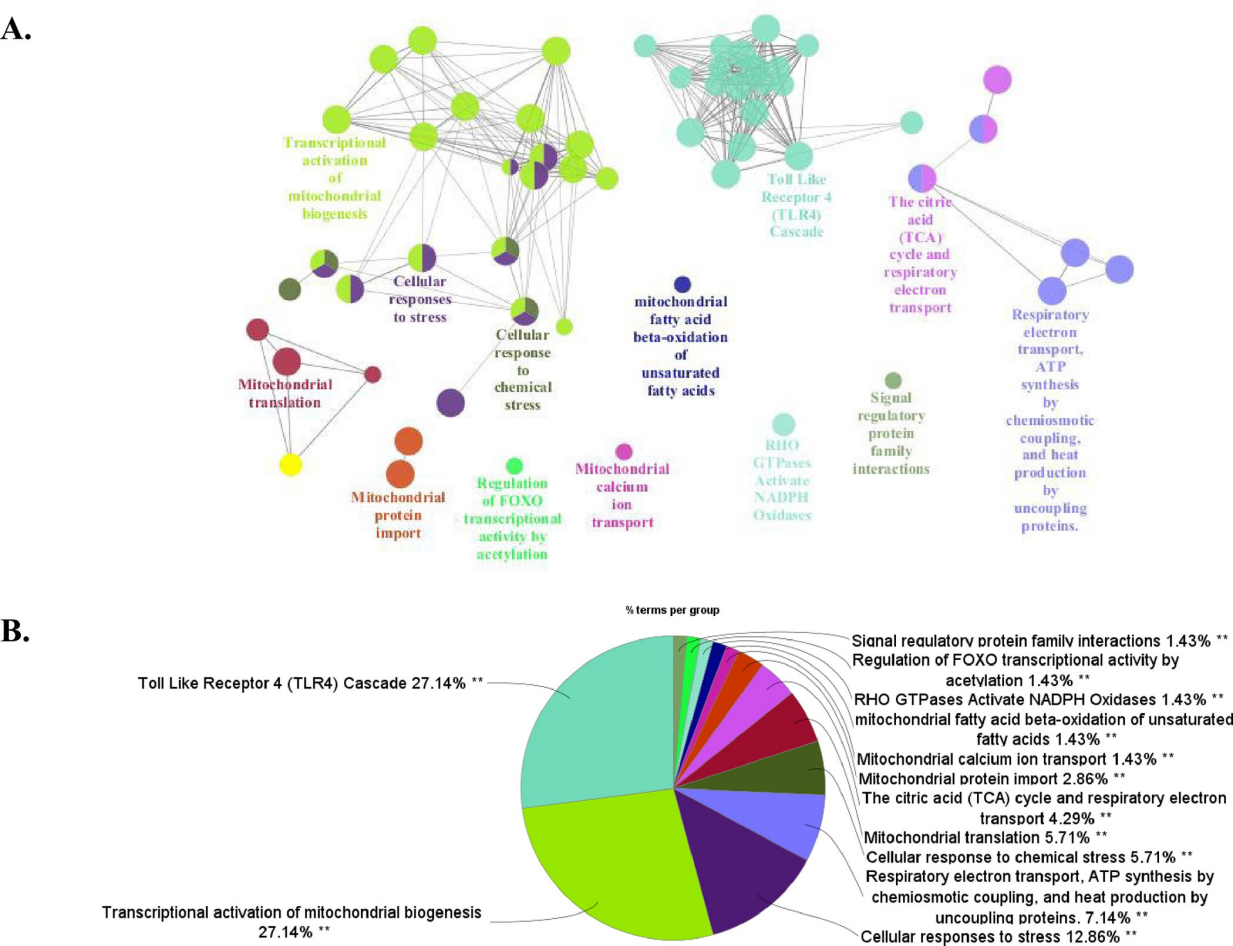
REACTOME pathway enrichment analysis of mitochondria-related immunometabolism. REACTOME pathway analysis was performed for differentially expressed genes in the microarray analysis of Cohort 1 using the newly generated mitochondria-related gene set. **A:** The interaction network of gene ontology (GO) terms in the metastasis-negative group compared with metastasis-positive group presented by ClueGO plug-in for Cytoscape software version 3.10.1 (https://cytoscape.org). GO terms describing molecular interactions among targets are represented as nodes, with node size representing the term enrichment significance. **B:** Pie chart showing the percentage of each GO term in the metastasis-negative group compared with metastasis-positive group.

To clarify the mitochondria-related pathways associated with the lymphatic spread of OPCs, REACTOME pathway analyses were performed for the 206 upregulated genes (Fig 1). REACTOME pathway analyses results are visualized in Fig 3A using ClueGO. The Toll-like receptor 4 (TLR4) cascade followed by transcriptional activation of mitochondrial biogenesis was the most significantly enriched REACTOME pathway for metastasis-negative cases (Fig 3B). Among the 206 upregulated genes, 13 (*APP*, *ATF2*, *BIRC2*, *BIRC3*, *CASP8*, *CREB1*, *ECSIT*, *MAP3K1*, *MAPK1*, *MAPK8*, *MAPK9*, *MEF2C*, and *TLR4*) were TLR4 cascade-related, and 21 (*ATF2*, *CHD9*, *CREB1*, *CREBBP*, *CRTC2*, *CRTC3*, *GABPB1*, *HCFC1*, *MED1*, *MEF2C*, *MEF2D*, *NCOA1*, *NCOA2*, *NCOA6*, *NCOR1*, *NR1D1*, *NRF1*, *PPRC1*, *SSBP1*, *TFB1M*, and *TGS1*) were related to transcriptional activation of mitochondrial biogenesis.

### Gene expression profile analyses at lymphoid ROIs by GeoMx WTA identified DEGs between metastasis-negative and metastasis-positive OPCs

In our previous work [11], DEG analysis of immune-related genes between metastasis-negative and metastasis-positive cases yielded greater sensitivity in lymphoid ROIs than in tumor ROIs for corresponding samples. In the current study, DEG analysis for mitochondria-related genes was also performed to compare their expression between lymphoid and tumor ROIs.

For Cohort 2, volcano plot analyses using the 948 mitochondria-related genes, described in Materials and Methods, illustrates the DEGs between metastasis-negative and metastasis-positive cases in lymphoid and tumor ROIs, respectively (Fig 2C and 2D). A total of 9 genes were upregulated in lymphoid ROIs, in contrast, no DEGs were identified in tumor ROIs.

These results suggest that lymphoid ROI analysis is more sensitive for detecting mitochondria-related DEGs between metastasis-negative and metastasis-positive cases than tumor ROI analysis from corresponding samples.

### Variant detection in mtDNA by matched pair analyses using NGS

As described above, no DEG was found in tumor ROIs between metastasis-negative and metastasis-positive cases using GeoMx WTA analysis. Additionally, tumor cell-related mtDNA sequence variations and their tumor-related functional changes were also examined using NGS.

mtDNA sequence variant detection was examined using matched pair samples of cancer nests and normal epithelia from Cohort 3 patients with OPC. From the analyses, a total of 5 variants from 3 patients were observed, all of which were single nucleotide polymorphisms (SNPs) (Table 3). These mutations were identified in the *ND1*, *ATP8*, and *CYB* regions, with an average variant allele frequency of 10.2% (1.40% - 30.30%). However, from the dbSNP database (https://www.ncbi.nlm.nih.gov/snp/), these mutations are not yet registered as malignancy related. These data, in addition to the data from the mitochondria-related DEG analysis using the GeoMx WTA assay for tumor ROIs described above, suggest that no lymph node metastasis-related OPC tumor cell-specific mtDNA variant and transcriptional change was observed using the tumor cell nest analyses.

**Table 3:** Mitochondrial DNA variants detected by matched pair analyses.

Patient Number	Position	Reference Allele	Variant Allele	Variant Allele Frequency	Mutation	Amino Acid Change	dbSNP rsID	Gene
1	3497	C	T	3.70%	amino acid substitution	p.Ala64Val	rs200319905	*ND1*
	3935	G	A	30.30%	amino acid substitution	p.Gly210Asp	N/A	*ND1*
2	8541	G	A	5.70%	amino acid substitution	p.Cys59Tyr	rs1569484218	*ATP8*
	15366	A	G	1.40%	amino acid substitution	p.Asn207Ser	N/A	*CYB*
4	3937	T	C	9.90%	amino acid substitution	p.Phe211Leu	N/A	*ND1*

SNP, single nucleotide polymorphism.

### Peritumoral tonsillar tissues of metastasis-negative OPCs are enriched in naïve B cells compared with metastasis-positive tissues

Following evaluation of the features of immunometabolism, immune cell alterations in the peritumoral tonsillar tissues of OPCs according to nodal status were examined.

**Fig 4:**
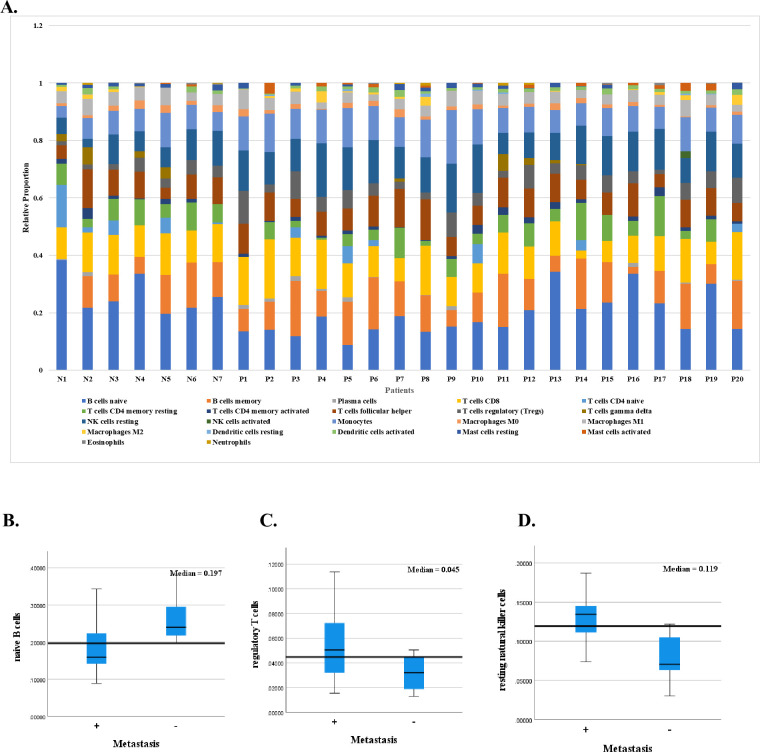
The relative immune cell proportions of 22 immune cell types in each sample from Cohort 1 estimated using the CIBERSORTx algorithm. **A:** N1-7 and P1-20 indicate each lymph node metastasis-negative and metastasis-positive patient, respectively. **B:** The relative proportion of naïve B cells in peritumoral tonsillar tissue was significantly higher in metastasis-negative oropharyngeal cancers (OPCs) compared with metastasis-positive (p = 0.009). **C** and **D:** In contrast, the relative proportions of regulatory T cells (Tregs) (p = 0.036) and resting natural killer (NK) cells (p = 0.002) were significantly lower in metastasis-negative OPCs compared with metastasis-positive. No significant difference was observed for the other 19 immune cell types. A p-value < 0.05 was considered significant by Mann-Whitney-U test. +, metastasis-positive; -, metastasis-negative.

The peritumoral tonsillar area microarray data from Cohort 1 samples were analyzed using the CIBERSORTx algorithm. Relative immune cell counts for each sample are illustrated in Fig 4A. The peritumoral tonsillar tissues of metastasis-negative OPC patients have a significantly higher rate of naïve B cells (p = 0.009), but lower for regulatory T cells (Tregs) (p = 0.036) and resting natural killer (NK) cells (p = 0.002), compared with those of metastasis-positive tissues (Fig 4B–4D).

## Discussion

In the present study, both the microarray analysis of peritumoral tonsillar tissues and the WTA analysis of lymphoid ROIs adjacent to tumor nests revealed a multitude of differentially expressed mitochondria-related genes according to OPC nodal status, irrespective of HPV status. Furthermore, REACTOME pathway analysis revealed significantly enriched pathways for metastasis-negative cases. Finally, the tonsillar tissues of metastasis-negative OPCs yielded significantly greater naïve B cell numbers, but fewer Tregs and resting NK cells. These results indicate that, as well as lymphoid tissues adjacent to tumor nests including tumor infiltrating lymphocytes (TILs) and tertiary lymphoid structures (TLSs), the peritumoral tonsillar tissues are a significant target for immunological investigation of OPC progression, focusing on immune cell counts and immunometabolism.

TILs are demonstrated prognostic biomarkers for many cancer types [16]. In head and neck cancer tumor tissues, it is reported that increased naïve B cell, Treg, and follicular helper T cell numbers are associated with improved outcomes [17]. Also associated with a favorable prognosis are increased numbers of CD8+ T cells and Tregs [18]. In OPCs, high CD4^+^ TILs are associated with significantly better overall survival, as well as high CD8^+^ TILs [19]. Although some reports analyzed TIL immune cell counts as above, no reports analyzed immune cell counts in the peritumoral tonsillar tissues of head and neck cancers. Therefore, this is the first report showing immune cell count changes in OPC peritumoral tonsillar tissues. In the current study, peritumoral tonsillar tissues of metastasis-negative patients had a significantly greater naïve B cell ratio as estimated from relative subsets of known RNA transcripts. B cells are particularly important in redirecting anticancer immune responses, as they may counteract the immunosuppressive environment of several cancers [20–22]. In head and neck cancers, especially HPV-positive cancers, greater plasma cell and memory B cell infiltration may indicate better prognosis [23,24]. However, the role of increased naïve B cell numbers in peritumoral tonsillar tissue, as well as in TILs and/or TLS, are not well characterized. Physiologically, naïve B cells are recruited from blood circulation into lymphoid organs, such as SLOs [9]. In the SLOs including tonsils, naïve B cells interact with follicular dendritic cells, which is critical for germinal center generation and maturation and enhancing tumor-specific immune-activity [11,20]. Thus, the naïve B cell increase in peritumoral tonsillar tissues is followed by an increased tumor-specific B cell influx into tumor tissues. Both the increase of naïve B cells and decrease of Tregs at the peritumoral tonsillar tissue would be advantageous for stimulating anti-tumor immune activities at tumor nests, resulting in lymph node metastases suppression in OPCs.

Cancer cells in a hypoxic TME exist at acidic pH, due to aerobic glycolysis promotion accompanied by lactate and H^+^ extracellular efflux [25]. A central metabolic program associated with innate and adaptive immune cell activation also employs aerobic glycolysis [6,7]. Therefore, highly proliferating tumor cells and their neighboring immune cells compete for glucose. Glucose deprivation due to glucose competition severely restricts immune cell cytotoxic activities [5]. Moreover, lactate release from tumor cells due to enhanced glucose use under aerobic glycolysis influences a wide range of immunosuppressive functions. Therefore, if tumor-infiltrating immune cells used lactate as a secondary energy source for mitochondrial respiration with extensive OXPHOS, these immune cells will be advantageous in exerting anti-tumor immunity. In the current study, REACTOME pathway analysis revealed the critical roles of some mitochondria-related pathways in OPC peritumoral tonsillar tissue for lymphatic dissemination suppression, regardless of HPV-status. This is the first report showing the significance of mitochondrial immunometabolism in peritumoral tonsillar tissues, the immune cell source for OPC tumor nests.

Peritumoral tonsillar tissue REACTOME pathway analyses revealed that 1) the TLR4 Cascade was the most significantly enriched pathway for metastasis-negative cases compared with metastasis-positive, followed by 2) the transcriptional activation of mitochondrial biogenesis. The TLR family consists of vital receptors responsible for pattern recognition in innate immunity, making them core proteins involved in pathogen detection and eliciting immune responses [26]. TLR4 is best known for the detection of gram-negative bacteria lipopolysaccharides (LPS) as a main pathogen-associated molecular pattern. It is reported that TLR4 cascade activation enhances both innate and adaptive immunity [27–29]. By LPS-induced TLR4 signaling activation, macrophages induce enhanced mitochondria-derived ROS production, which increases macrophage bactericidal ability [27,29]. TLR4 cascade-activated DCs induce NF-κB and NF-κB-controlled genes for inflammatory cytokines, as well as co-stimulatory molecules CD80 and 86 expression, which are required for naïve T cell activation [28]. Furthermore, patients with OPCs, especially in HPV-positive cases, have a microbiome shifted toward gram-negative bacteria with LPS, indicating greater TLR4-activation susceptibility [30]. Thus, in peritonsillar tissues with the TLR4 cascade upregulated, both the innate and adaptive immune systems are activated, enhancing inflammation, supplying the primary site with tumor-specific immune cells from the peritumoral tonsillar tissue, and finally suppressing OPC lymph node metastasis.

Mitochondrial biogenesis, fusion, and fission are involved in immune cell activation [31]. These dynamics regulate OXPHOS and immunity, and this is evident in T cells. Fused elongated mitochondria tend to have efficient electron-transport chain supercomplex formation and OXPHOS, assisting in cell survival [32]. Fission generates fragmentated mitochondria with increased ROS production, which is important during effecter T cell activation [33]. Activated naïve T cells rapidly increase mitochondrial mass, mitochondrial respiration, and mitochondrial ROS generation, which are inter-linked and important for effecter T cell maturation [34]. Thus, mitochondrial biogenesis pathways are involved in the suppression mechanism of OPC lymph node metastases.

Although our mitochondria-related gene-set-based data are encouraging for clarification of the peritumoral tonsillar tissue immunometabolic background, and shows promise for lymph node metastasis prediction and possible regulation, limitations to this study exist. First, the analyzed cohorts were from retrospective samples from our pathology files with limited sample numbers, especially in Cohort 2 for GeoMx WTA and in Cohort 3 for mtDNA NGS. However, GeoMx WTA and mtDNA NGS were performed to confirm the consistent lack of statistical significance observed for tumor tissue-related genes in the lymphatic progression of OPCs. Therefore, as the next step of this study, prospective result validation is necessary on a priority basis in peritumoral tonsillar tissue analyses from prospective and multi-institutional studies. Second, the mitochondria-related pathway results were estimated from the transcriptomic data. Thus, the correlation between our signatures, such as the TLR4 cascade and mitochondrial biogenesis, and data from microbiome and mitochondrial dynamics, still warrants verification by future studies with both *in vitro* and *in vivo* analyses.

In summary, the current analyses identified mitochondria-related transcriptional programs associated with lymph node metastases as well as immune cell profiles in the peritumoral tonsillar tissues of OPCs. Further evaluation of peritumoral tonsils will elucidate targetable immune mechanisms associated with OPC lymphatic dissemination.

## Supporting information

S1 Fig Detection of differentially expressed genes (DEGs) in metastasis-negative (Met-) and metastasis-positive (Met+) oropharyngeal cancers (OPCs) in microarray analysis. **A:** An example of peri-tumoral tonsillar tissue macro-dissection from a lateral oropharyngectomy surgical specimen. ▲, tumor tissue; ★, peritumoral tonsillar tissue. The red frame indicates the peritumoral tonsillar specimen area obtained by macro-dissection for microarray analysis. **B:** Volcano plot of DEGs in Met- and Met+ OPCs in the microarray analyses of Cohort 1. **Red** and **green dots** indicate up- and downregulated genes in Met- cases compared with Met+ cases, respectively. Analyses of OPC peri-tumoral tonsillar samples revealed 206 upregulated and 22 downregulated genes. Statistical significance was defined as |log_2_| of fold change > 1 and -log_10_ p-value > 1.3.
(TIF)

S1 Table List of the newly generated mitochondria-related gene set. (XLSX)
