# Supplementary figures and images for: Lymph node metastasis regulation by peritumoral tonsillar tissue mitochondria-related pathway activation in oropharyngeal cancer
Source: PLoS One. 2024 Feb 28;19(2):e0299750. doi: 10.1371/journal.pone.0299750 (PMC10901332; doi:10.1371/journal.pone.0299750)

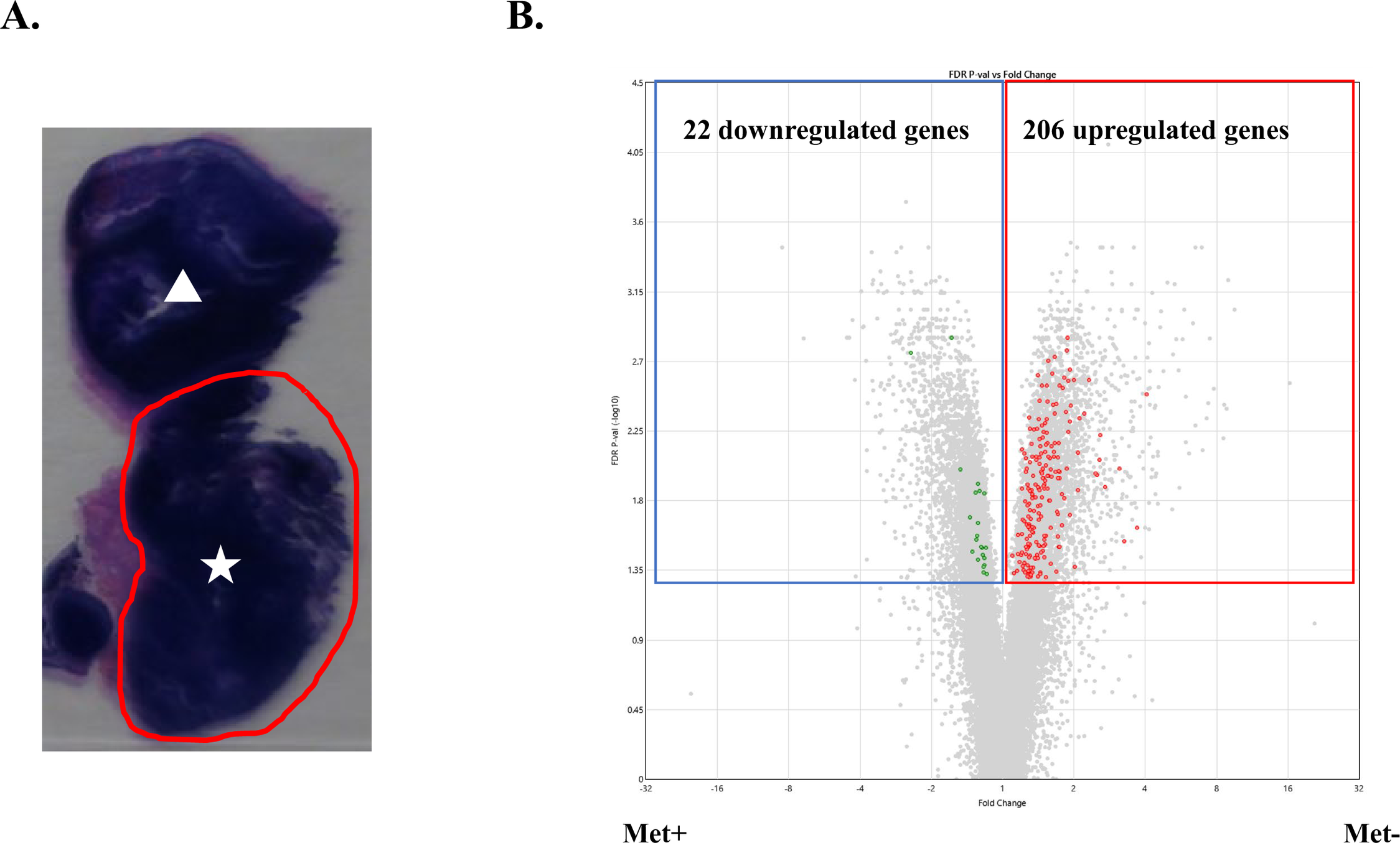

Supplement: S1 Fig — A: An example of peri-tumoral tonsillar tissue macro-dissection from a lateral oropharyngectomy surgical specimen. ▲, tumor tissue; ★, peritumoral tonsillar tissue. The red frame indicates the peritumoral tonsillar specimen area obtained by macro-dissection for microarray analysis. B: Volcano plot of DEGs in Met- and Met+ OPCs in the microarray analyses of Cohort 1. Red and green dots indicate up- and downregulated genes in Met- cases compared with Met+ cases, respectively. Analyses of OPC peri-tumoral tonsillar samples revealed 206 upregulated and 22 downregulated genes. Statistical significance was defined as |log2| of fold change > 1 and -log10 p-value > 1.3. (TIF) [file pone.0299750.s001.tif]
